# SPDEF suppresses head and neck squamous cell carcinoma progression by transcriptionally activating NR4A1

**DOI:** 10.1038/s41368-021-00138-0

**Published:** 2021-10-20

**Authors:** Yanting Wang, Xianyue Ren, Weiyu Li, Ruoyan Cao, Suyang Liu, Laibo Jiang, Bin Cheng, Juan Xia

**Affiliations:** 1 Hospital of Stomatology, Sun Yat-sen University, Guangzhou, China; 2 Guangdong Provincial Key Laboratory of Stomatology, Guangzhou, China; 3 Guanghua School of Stomatology, Sun Yat-sen University, Guangzhou, China

**Keywords:** Oncology, Cancer

## Abstract

SAM pointed domain containing E26 transformation-specific transcription factor (SPDEF) plays dual roles in the initiation and development of human malignancies. However, the biological role of SPDEF in head and neck squamous cell carcinoma (HNSCC) remains unclear. In this study, the expression level of SPDEF and its correlation with the clinical parameters of patients with HNSCC were determined using TCGA-HNSC, GSE65858, and our own clinical cohorts. CCK8, colony formation, cell cycle analysis, and a xenograft tumor growth model were used to determine the molecular functions of SPDEF in HNSCC. ChIP-qPCR, dual luciferase reporter assay, and rescue experiments were conducted to explore the potential molecular mechanism of SPDEF in HNSCC. Compared with normal epithelial tissues, SPDEF was significantly downregulated in HNSCC tissues. Patients with HNSCC with low SPDEF mRNA levels exhibited poor clinical outcomes. Restoring SPDEF inhibited HNSCC cell viability and colony formation and induced G0/G1 cell cycle arrest, while silencing SPDEF promoted cell proliferation in vitro. The xenograft tumor growth model showed that tumors with SPDEF overexpression had slower growth rates, smaller volumes, and lower weights. SPDEF could directly bind to the promoter region of NR4A1 and promoted its transcription, inducing the suppression of AKT, MAPK, and NF-κB signaling pathways. Moreover, silencing NR4A1 blocked the suppressive effect of SPDEF in HNSCC cells. Here, we demonstrate that SPDEF acts as a tumor suppressor by transcriptionally activating NR4A1 in HNSCC. Our findings provide novel insights into the molecular mechanism of SPDEF in tumorigenesis and a novel potential therapeutic target for HNSCC.

## Introduction

As the sixth most common cancer in the world,^1^ the global incidence of head and neck squamous cell carcinoma (HNSCC) is ~600 000 per year, with over 300 000 deaths.^2^ Despite improvements in current surgery and adjuvant therapies, the 5-year survival rate of patients with HNSCC is only ~50%.^3^ Major risk factors include tobacco, alcohol, and human papillomavirus.^4^ However, the molecular mechanisms underlying HNSCC occurrence remain unclear^5,6^ and identifying novel therapeutic targets may improve HNSCC treatment outcomes.^7^

The E26 transformation-specific (ETS) proteins are a family of transcription factors characterized by an evolutionarily conserved DNA-binding domain that controls important cellular processes such as DNA repair, cell cycles, cytoskeleton organization, and tRNA biosynthesis.^8,9^ SAM pointed domain containing E26 transformation-specific transcription factor (SPDEF) is a member of the ETS family and plays crucial roles in physiological and pathological processes, including in the occurrence and development of cancers.^10,11^ The expression levels and molecular functions of SPDEF vary in different malignant tissues. SPDEF is overexpressed and promotes the occurrence and metastasis of gastric cancer,^12,13^ whereas it can inhibit prostate cancer cell migration and invasion.^11,14^ In colorectal cancer cells, SPDEF induces a quiescent state by disrupting β-catenin binding to cell cycle-related genes.^15,16^ Thus, SPDEF has complex roles in different cancers. However, the biological significance and underlying mechanisms of SPDEF in HNSCC remain unknown.

Nuclear Receptor Subfamily 4 Group A Member 1 (NR4A1, also known as TR3, Nur77, or NGFI-B), which acts as a transcription factor, is a nuclear orphan receptor belonging to the subfamily of NR4A nuclear receptors.^17^ NR4A1 is induced by diverse cellular signals and transcriptionally activates different genes, including those involved in cell differentiation, proliferation, cell cycle arrest, and apoptosis.^18,19^ Previous studies have reported that NR4A1 is downregulated and suppresses tumor progression by suppressing AKT, MAPK, NF-κB, WNT, and JNK/Parkin signaling pathways in several cancers, including colon cancer, breast cancer, and oral squamous cell carcinoma.^20–25^ The molecular events associated with NR4A1 downregulation have, however, not been fully elucidated.

In this study, we examined the biological role of SPDEF in HNSCC progression and its relationship with clinical parameters in patients with HNSCC. We revealed that the downregulation of SPDEF was associated with poor overall survival in patients with HNSCC. SPDEF suppressed HNSCC cell proliferation in vitro and in vivo. The underlying mechanism was attributed to the transcriptional activation of NR4A1, which ultimately suppressed AKT, MAPK, and NF-κB signaling pathways. In summary, our findings demonstrate a novel SPDEF regulation mechanism that governs HNSCC tumorigenicity and provides important insights into targeted therapies for patients with HNSCC.

## Results

### SPDEF downregulation is associated with poor survival in HNSCC

**Fig. 1:**
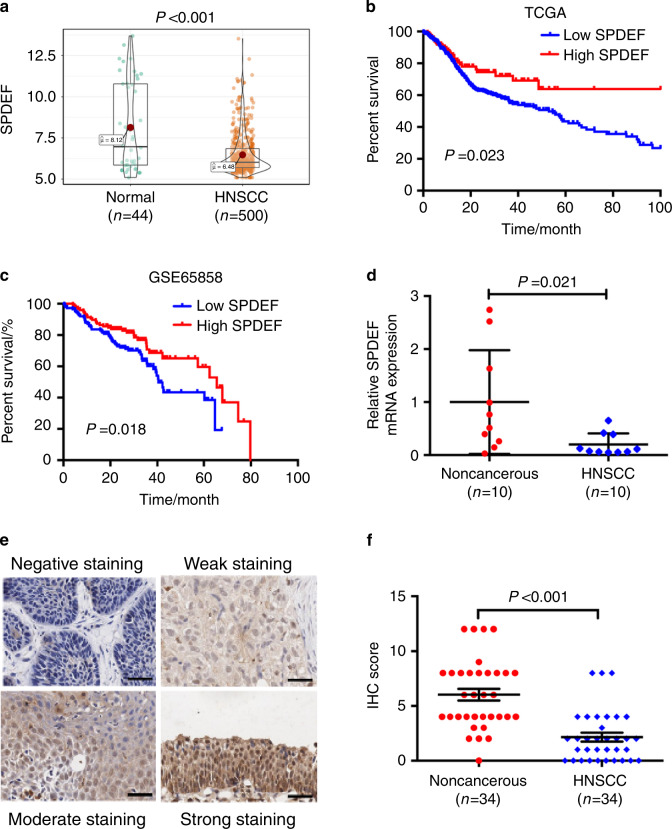
Downregulation of SPDEF is associated with poor overall survival in HNSCC. **a** SPDEF mRNA levels in normal (*n* = 44) and HNSCC (*n* = 500) tissues were determined using the HNSC cohort from TCGA. **b**, **c** Kaplan–Meier method was used to analyze overall survival based on SPDEF mRNA levels (Low SPDEF versus High SPDEF) in patients with HNSCC in TCGA (*n* = 500) (**b**) and GSE65858 (*n* = 270) (**c**) cohorts. **d** SPDEF mRNA levels in non-cancerous (*n* = 10) and HNSCC (*n* = 10) tissues were examined using real-time RT-PCR analysis. **e**, **f** The representative images (×200) (**e**) and quantification analysis (**f**) of SPDEF protein levels in non-cancerous (*n* = 34) and HNSCC (*n* = 34) tissues were assessed by immunohistochemical staining. Scale bar: 40 µm. Error bars, mean ± SD

We analyzed the SPDEF mRNA levels in a HNSCC cohort in The Cancer Genome Atlas (TCGA) database. The results showed that the expression levels of SPDEF were significantly lower in HNSCC tissues (*n* = 500) compared with normal epithelial tissues (*n* = 44) (*P* < 0.001, Fig. 1a). The optimal cutoff value (6.963) stratified the patients into high (*n* = 115) and low (*n* = 385) SPDEF expression groups. Kaplan–Meier survival analysis indicated that the overall survival times of HNSCC patients with low SPDEF mRNA levels were shorter than those of patients with high SPDEF mRNA levels (*P* = 0.023, Fig. 1b). Patients from a separate HNSCC dataset (GSE65858) were divided into high (*n* = 124) or low (*n* = 146) SPDEF expression groups based on the optimal cutoff value (6.416), and similar results were obtained (*P* = 0.018, Fig. 1c). Hence, these data demonstrate that SPDEF can serve as a potential prognostic indicator for HNSCC.

Next, the relationship between SPDEF and clinical parameters of patients with HNSCC was examined using our own clinical cohort. Real-time RT-PCR and immunohistochemistry (IHC) assays were performed to determine the mRNA and protein levels of SPDEF in paired primary HNSCC tumors and adjacent non-cancerous epithelial tissues. Lower SPDEF mRNA (*n* = 10, *P* = 0.021, Fig. 1d) and protein (*n* = 34, *P* < 0.001, Fig. 1e, f) levels were observed in tumor tissues compared with the adjacent non-cancerous tissues. Furthermore, we divided these 34 patients into high (*n* = 17) and low (*n* = 17) SPDEF expression groups based on the median value of the staining index (SI) (SI = 2). The results showed that lower SPDEF protein levels were associated with more advanced tumor phenotypes, including primary tumor size (*P* = 0.034), regional lymph node metastasis (*P* = 0.019), and tumor node metastasis (TNM) stage (*P* = 0.019, Table 1). In GSE65858 cohort, lower SPDEF mRNA levels were also associated with more advanced T stages (*P* = 0.003, Table S1). However, in TCGA-HNSC cohort, no significant correlations were observed between SPDEF mRNA levels and patients’ clinical features, implying heterogeneity in different patient populations. Collectively, these results demonstrate that SPDEF is downregulated in HNSCC, and its low expression pattern is correlated with advanced phenotype and poor clinical outcome.

**Table 1:** Relationship between SPDEF protein levels and clinicopathologic characteristics of patients with HNSCC

Variables	SPDEF expression (*n* = 34)			
	Low/%	High/%	Total	*P* value^a^
Age				
<60 years	10 (52.6)	9 (47.4)	19	0.470
≥60 years	6 (40)	9 (60)	15	
Gender				
Male	13 (48.1)	14 (51.9)	27	0.806
Female	3 (42.9)	4 (57.1)	7	
T stage				
T1–T2	9 (36)	16 (64)	25	0.034^*^
T3–T4	7 (77.8)	2 (22.2)	9	
N stage				
N_0_	5 (27.8)	13 (72.2)	18	0.019^*^
N_1_–N_3_	11 (68.8)	5 (31.3)	16	
TNM stage				
I–II	5 (27.8)	13 (76.5)	18	0.019^*^
III–IV	11 (68.8)	5 (31.3)	16	

^a^*χ*^2^ test, ^*^P < 0.05

### SPDEF inhibits the proliferation of HNSCC cells in vitro

**Fig. 2:**
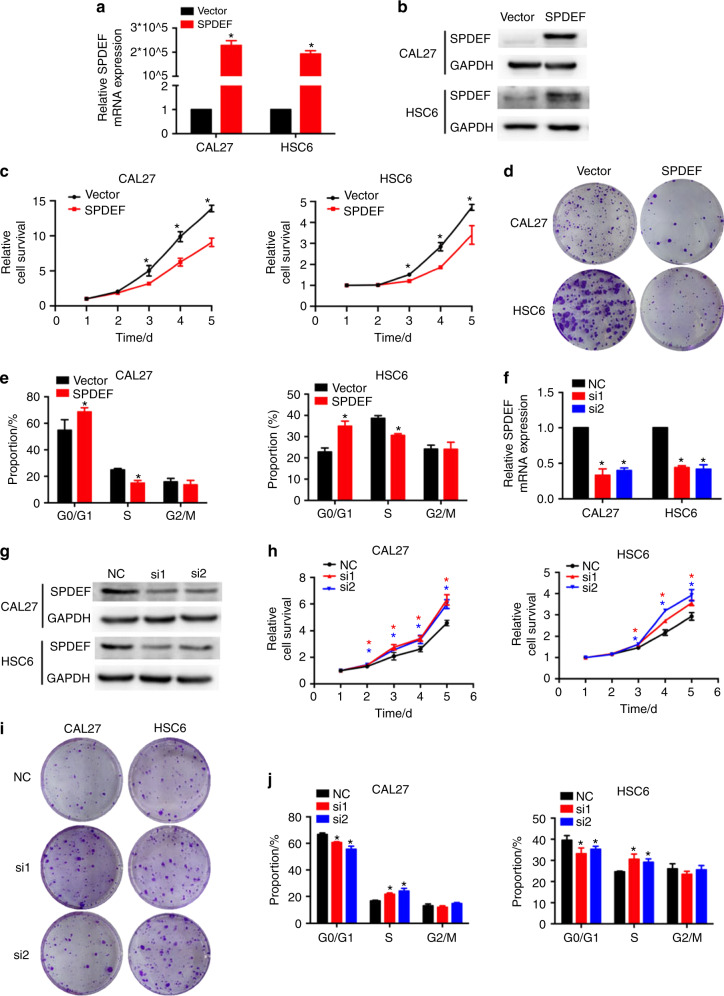
SPDEF suppresses HNSCC cell proliferation in vitro. **a**–**e** CAL27 and HSC6 cells stably overexpressing vector or SPDEF were used to determine the effects of SPDEF overexpression on HNSCC cell proliferation. SPDEF mRNA and protein levels were measured using real-time RT-PCR (**a**) and western blotting assays (**b**), respectively. Cell viability was measured using CCK-8 assay (**c**). The representative images of colonies were determined using the colony formation assay (**d**). The cell cycle was measured using flow cytometry (**e**) (*n* = 3). **f**–**j** CAL27 and HSC6 cells transiently transfected with control (NC) or SPDEF-siRNAs (si-1 and si-2) were used to determine the effects of silencing SPDEF on HNSCC cell proliferation. The SPDEF mRNA and protein levels were measured using real-time RT-PCR (**f**) and western blotting assays (**g**), respectively. Cell viability was measured using CCK-8 assay (**h**). The representative images (**i**) of colonies were determined using the colony formation assay. The cell cycle was measured using flow cytometry (**j**) (*n* = 3). Error bars, mean ± SD; ^*^P < 0.05

We constructed CAL27 and HSC6 cells that stably overexpressed vectors or SPDEF to explore the effect of SPDEF on HNSCC progression. Transfection efficiency was examined using real-time RT-PCR and western blotting assays. The results showed that, in contrast to cells with vector overexpression, the mRNA and protein levels of SPDEF were obviously elevated in SPDEF-overexpressing cells (Figs. 2a, b and S1a). Restoring SPDEF subsequently inhibited the viability and colony formation abilities of HNSCC cells (Figs. 2c, d and S2a). Furthermore, more cells were arrested in the G0/G1 cell cycle in the SPDEF-overexpressing group than in the vector-overexpressing group (Fig. 2e).

The small interfering RNAs (siRNAs) of SPDEF (si-1 and si-2) were used to silence SPDEF expression in CAL27 and HSC6 cells. Real-time RT-PCR and western blotting assays confirmed that the siRNAs could significantly reduce the mRNA and protein levels of SPDEF in both cell lines (Figs. 2f, g and S1b). CCK8 and colony formation assays showed that knocking down SPDEF promoted the viability and colony formation abilities of CAL27 and HSC6 cells (Figs. 2h, i and S2b). In addition, silencing SPDEF reduced G0/G1 cell cycle arrest in CAL27 and HSC6 cells (Fig. 2j).

### SPDEF suppresses AKT, MAPK, and NF-κB signaling pathways in HNSCC

**Fig. 3:**
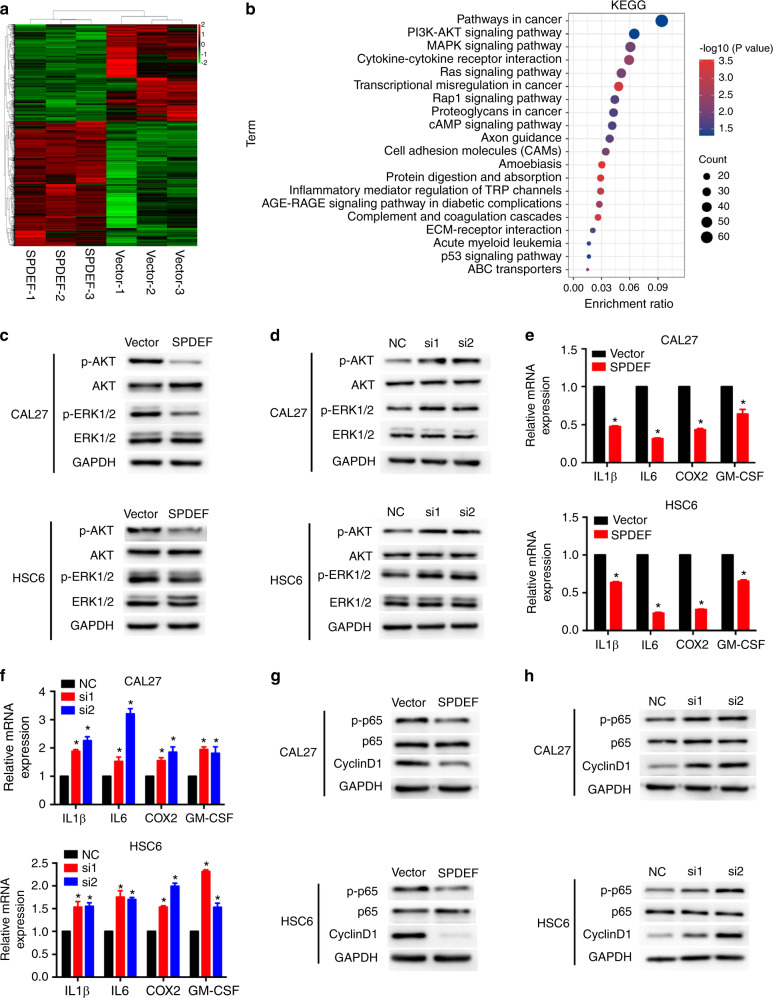
SPDEF suppresses AKT, MAPK, and NF-κB signaling pathways in HNSCC. **a** Heatmap of differently expressed genes in CAL27 cells overexpressing SPDEF compared with cells overexpressing vector. *P* < 0.05, fold change value >1.5. **b** KEGG analysis of differently expressed genes in CAL27 cells overexpressing SPDEF compared with cells overexpressing vector. **c**–**h** CAL27 and HSC6 cells overexpressing SPDEF and CAL27 and HSC6 cells in which SPDEF was silenced were used to explore the effects of SPDEF on AKT, MAPK, and NF-κB signaling in HNSCC cells. Western blotting analysis of p-AKT, AKT, p-ERK1/2, and ERK1/2 protein levels. GAPDH was used as an endogenous control (**c**, **d**). The NF-κB target gene (IL1β, IL6, COX2, GM-CSF) mRNA levels were determined by real-time RT-PCR analysis (**e**, **f**). Western blotting analysis of p-p65, p65, and cyclinD1 protein expression. GAPDH was used as an endogenous control (**g**, **h**). Error bars, mean ± SD; ^*^P < 0.05

To confirm the downstream effects of SPDEF, we performed a global transcriptome analysis using RNA sequencing (RNA-seq) in CAL27 cells stably overexpressing vector or SPDEF. A total of 2 408 genes were differently expressed between the vector and SPDEF groups (Fig. 3a). Based on results from the Kyoto Encyclopedia of Genes and Genomes (KEGG) analysis, differently expressed genes were mainly enriched in pathways associated with cancer progression, including the PI3K/AKT and MAPK signaling pathways (Fig. 3b). Western blotting assays revealed that the expression levels of p-AKT, p-GSK3β, and p-ERK1/2 were lower in SPDEF-overexpressing cells than in vector-overexpressing cells (Figs. 3c; S3a and S4a, b). P-APK, p-GSK3β, and p-ERK1/2 expression levels were restored in CAL27 and HSC6 cells when SPDEF was silenced (Figs. 3d; S3b and S4c, d).

Several immune-associated genes in our RNA-seq dataset were dysregulated by SPDEF, including genes enriched in inflammatory mediator regulation of TRP channels, cytokine-cytokine receptor interaction, complement and coagulation cascades (Figs. 3b and S5a). Given that the NF-κB signaling pathway plays critical roles in immune response regulation and tumor cell formation,^26^ we explored alterations in genes in the NF-κB signaling pathway in SPDEF-overexpressing cells. IL-1β, IL-6, COX2, and GM-CSF, the NF-κB signaling target genes, were substantially downregulated in SPDEF-overexpressing cells, which was verified by real-time RT-PCR (Figs. 3e and S5b). In CAL27 and HSC6 cells in which SPDEF was silenced, the mRNA levels of IL-1β, IL-6, COX2, and GM-CSF were upregulated (Fig. 3f). The levels of phospho-p65 and cyclinD1 proteins were decreased in SPDEF-overexpressing cells and increased in SPDEF-silenced cells (Figs. 3g, h and S6). These data demonstrate that SPDEF might regulate HNSCC tumorigenicity by suppressing the AKT, MAPK, and NF-κB signaling pathways.

### SPDEF directly activates NR4A1 transcription in HNSCC

**Fig. 4:**
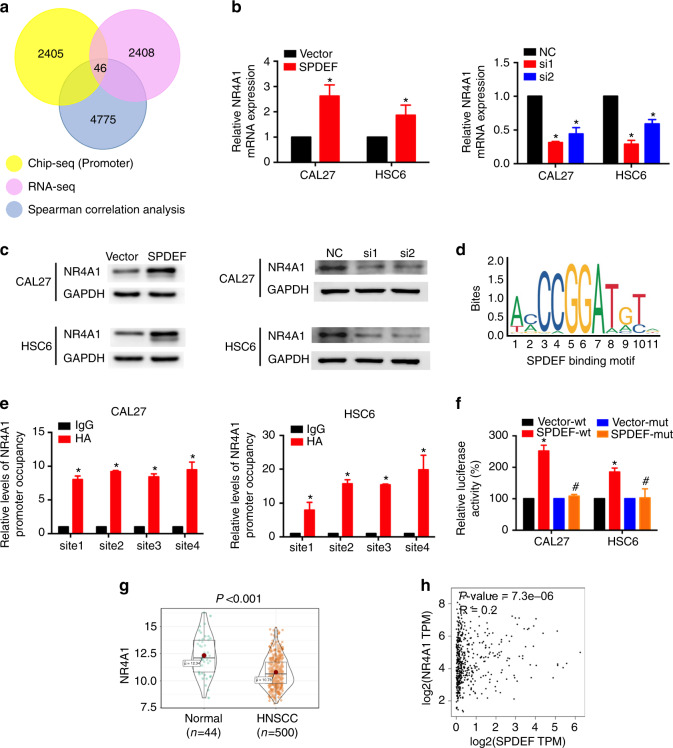
SPDEF directly activates NR4A1 transcription in HNSCC. **a** Venn diagram indicating the number of genes identified by ChIP-seq (GSE86957, promoter), RNA-seq, and Spearman correlation analysis (TCGA-HNSC cohort, *n* = 500). **b**, **c** NR4A1 mRNA and protein levels were detected using real-time RT-PCR (**b**) and western blotting assays (**c**), respectively. **d** The binding motif of SPDEF was predicted using the JASPAR database. **e** ChIP-qPCR was performed to assess the enrichment of SPDEF in the NR4A1 promoter region in HNSCC cells overexpressing SPDEF. **f** CAL27 and HSC6 cells were transfected with the pGL4-NR4A1-wild type or the pGL4-NR4A1-mutant promoter to determine the transcriptional activation efficiencies of SPDEF. Relative transcriptional activities were determined using dual luciferase reporter assay. ^*^P < 0.05 compared with Vector-wt; ^#^compared with SPDEF-wt. **g** NR4A1 mRNA levels of normal (*n* = 44) and HNSCC (*n* = 500) tissues in TCGA-HNSC cohort. **h** Correlation between SPDEF and NR4A1 mRNA levels in TCGA-HNSC cohort (*n* = 500) was assessed using Spearman correlation analysis. Error bars, mean ± SD; ^*^P < 0.05

SPDEF acts as a transcription factor by binding to the promoter regions of genes, thus we analyzed public SPDEF ChIP-seq data (GSE86957) to identify its potential target genes.^27^ We also performed Spearman correlation analysis using TCGA-HNSC cohort to determine genes that were positively correlated with SPDEF in HNSCC. A total of 46 differently expressed genes from our RNA-seq data were positively correlated with SPDEF in HNSCC, the promoter of which might bind to SPDEF (Fig. 4a). Among the top 20 genes, NR4A1, which is known to regulate cancer cell proliferation by suppressing AKT, MAPK, and NF-κB signaling pathways, was upregulated in SPDEF-overexpressing cells (Table S2).^18–20^ Real-time RT-PCR and western blotting assays verified that overexpressing SPDEF could increase NR4A1 mRNA and protein expression levels, while knocking down SPDEF had the opposite effect (Figs. 4b, c and S7).

As a transcription factor, the high-affinity sequence of GGAT has been identified as the binding motif of SPDEF.^12^ The JASPAR database was used to predict the potential binding sites of SPDEF on the proximal promoter of NR4A1, as previously reported.^28^ In total, four potential candidate binding sites were identified in the proximal promoter region of NR4A1 (Fig. 4d and Table S3). We then performed chromatin immunoprecipitation quantitative PCR (ChIP-qPCR), which showed that SPDEF could bind to all four candidate sites in CAL27 and HSC6 cells with exogenous SPDEF expression (Fig. 4e). To further examine the transcription activity of SPDEF on NR4A1, we amplified the NR4A1 proximal promoter region (2000 bp upstream of the transcription start site) and cloned it into a luciferase reporter vector (pGL4-basic). The binding site mutant NR4A1-Luc reporter was also generated. The dual luciferase reporter assay indicated that overexpression of SPDEF could subsequently activate the transcriptional activity of the wild-type NR4A1 promoter other than the mutant type (Fig. 4f).

Next, we determined the expression levels of NR4A1 and its correlation with SPDEF expression levels in HNSCC clinical tissues. In TCGA-HNSC cohort, we found that the mRNA levels of NR4A1 were lower in HNSCC tissues compared with normal tissues (*P* < 0.001, Fig. 4g). Furthermore, a positive correlation between SPDEF and NR4A1 mRNA levels was observed in HNSCC tumors (*P* = 7.3e-6, *R* = 0.2, Fig. 4h). In our hospital cohort, similar results were observed at both mRNA and protein levels (Fig. S8). Overall, these results demonstrate that SPDEF promotes NR4A1 transcription by directly binding to its promoter.

### NR4A1 is a functional target of SPDEF in HNSCC

**Fig. 5:**
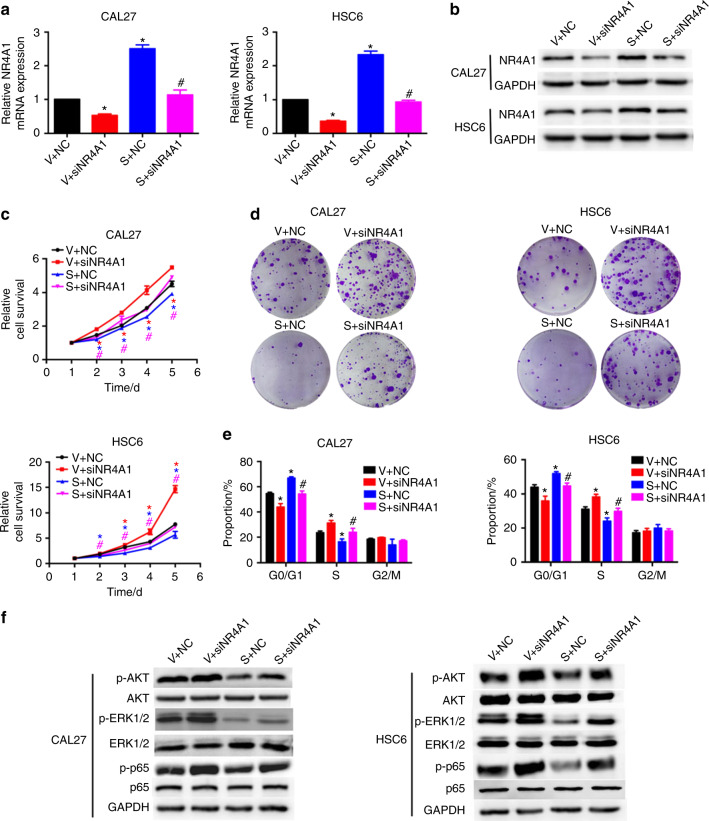
NR4A1 is a functional target of SPDEF in HNSCC. Negative Control (NC) or NR4A1-siRNA (siNR4A1) was transiently transfected into CAL27 and HSC6 cells stably overexpressing vector (V) or SPDEF (S). **a**, **b** NR4A1 mRNA and protein levels were measured using real-time RT-PCR (**a**) and western blotting assays (**b**), respectively. **c** Cell viability was measured using CCK-8 assay. **d** Colony formation ability was measured using colony formation assay. **e** The cell cycle was determined using flow cytometry. **f** The activities of AKT, MAPK, and NF-κB signaling pathways were measured using western blotting assays. Error bars, mean ± SD; ^*^P < 0.05 compared with V + NC; ^#^compared with S + NC

To further verify whether SPDEF-mediated NR4A1 upregulation contributed to the suppression of HNSCC cell proliferation, we silenced NR4A1 expression using siRNA in HNSCC cells stably overexpressing vector or SPDEF. The silencing efficiencies of NR4A1 mRNA and protein expression are shown in figures (Figs. 5a, b and S9). CCK-8 assays indicated that co-transfection with siNR4A1 significantly reversed the inhibitory effects of SPDEF on HNSCC cell viability (Fig. 5c). In addition, silencing NR4A1 also restored the inhibitory effects of SPDEF on colony formation and suppressed G0/G1 cell cycle arrest (Figs. 5d, e and S10). Furthermore, SPDEF’s inhibitory effects on the activities of AKT, MAPK, and NF-κB signaling pathways were restored after silencing NR4A1 (Figs. 5f and S11). Collectively, these findings illustrate that SPDEF can suppress HNSCC cell progression through NR4A1 activation.

### SPDEF inhibits HNSCC tumorigenesis in vivo

**Fig. 6:**
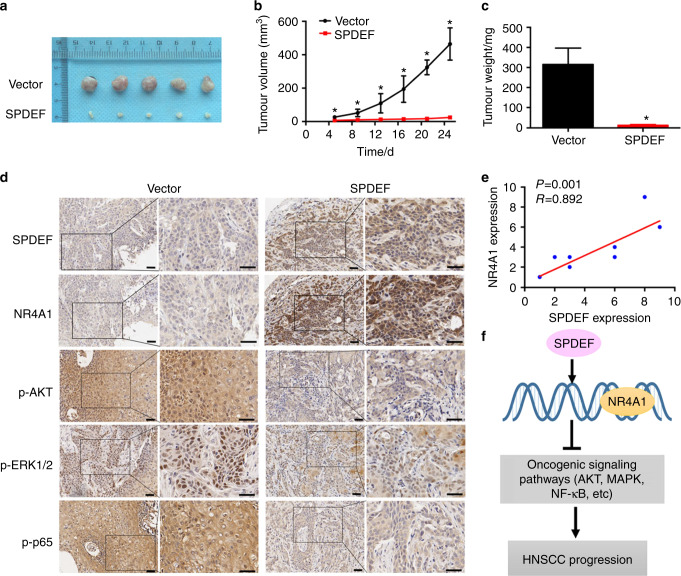
SPDEF inhibits HNSCC tumor growth in vivo. CAL27 cells stably overexpressing SPDEF or vector were subcutaneously injected into the flank of BALB/c-nu mice (*n* = 5). **a** Tumor nodules, **b** growth curves of tumor volume (**c**) and tumor weights were measured in mice xenografts. **d** The representative images (×100 and ×200) of SPDEF and NR4A1 protein levels in mice xenografts were assessed by immunohistochemical staining. Scale bar: 40 µm. **e** Correlation between SPDEF and NR4A1 protein levels in mice xenografts (*n* = 10) was measured using Spearman correlation analysis. **f** Schematic summary of the SPDEF-NR4A1-oncogenic signaling pathways. SPDEF suppresses HNSCC progression by directly promoting NR4A1 transcription and subsequently inactivating AKT, MAPK, and NF-κB signaling pathways. Error bars, mean ± SD; ^*^P < 0.05

A xenograft tumor model was developed to assess the effects of SPDEF on HNSCC cell growth in vivo. In contrast to tumors with vector overexpression group, the results showed that tumors with SPDEF overexpression group showed significantly smaller volumes, slower growth rates, and lower weights (Fig. 6a–c). IHC assays indicated that tumors with SPDEF overexpression group had higher levels of SPDEF and NR4A1 and lower AKT, MAPK, and NF-κB signaling pathway activities than those from the vector group (Figs. 6d and S12). IHC staining of xenograft tumors also showed a positive correlation between SPDEF and NR4A1 expression level (*P* = 0.001, *R* = 0.892, Fig. 6e). Hence, these findings demonstrate that SPDEF can promote NR4A1 expression and inhibit HNSCC cell growth in vivo.

## Discussion

In this study, we demonstrated that SPDEF is downregulated in HNSCC, and its low expression is correlated with poor survival status. Overexpression of SPDEF suppressed HNSCC cell viability, colony formation ability, and induced G0/G1 cell cycle arrest, while knocking down SPDEF exhibited the opposite outcomes in vitro. The subcutaneous xenogeneic model confirmed that SPDEF inhibited HNSCC tumor growth in vivo. Moreover, we demonstrated that SPDEF suppressed HNSCC cell progression by promoting NR4A1 transcription and inhibiting the activation of AKT, MAPK, and NF-κB signaling pathways. Hence, our results offer novel insights into the potential mechanisms of SPDEF in controlling HNSCC cell progression.

Many factors affect the clinical outcomes of patients with HNSCC, including late-stage diagnosis and unclear cancer progression mechanisms.^5,6^ Whole-genome transcriptome analysis has cataloged multiple gene expression alterations in HNSCC tumorigenesis whose effects remain unclear and need to be determined.^29^ Dysregulated transcription factors represent a unique class of the most direct and possible targets that mediate aberrant gene expression, including blockage of differentiation and hallmark features of cancers.^30^ In this study, we identified that SPDEF, a member of the ETS transcription factor family, is downregulated in HNSCC tissues and may play a protective role in HNSCC development.

An increasing number of studies show dysregulated SPDEF expression in human cancers, which are associated with different clinical features such as TNM stage, tumor grade, and survival.^11^ In ovarian cancer, early stage and borderline tumors expressed higher SPDEF than late-stage tumors. Patients with ovarian cancer showing positive SPDEF expression presented good prognosis.^31^ A multiple-cohorts analysis of prostate cancer demonstrated an inverse relationship between SPDEF expression and tumor progression and poor patient survival.^32^ However, high SPDEF levels have been linked to poor survival in estrogen receptor-positive breast cancer.^33^ In gastric cancer, SPDEF levels were higher in tumors than in peritumoral tissues.^13^ In this study, we explored the predictive effects of SPDEF on the overall survival of patients with HNSCC using two public cohorts and found that patients with low SPDEF exhibited poor clinical outcomes and advanced phenotypes, indicating that SPDEF might be a promising prognostic biomarker for HNSCC.

SPDEF plays critical roles in many biological processes by directly activating downstream target gene transcription. SPDEF could bind to MyD88 and TRIF and subsequently inhibited Toll-like receptor and type I interferon signaling during airway mucous metaplasia.^34^ In intestinal epithelium development, SPDEF regulated a specific subset of genes, including Cryptdins, Ang4, and Muc2, thus promoting terminal differentiation of secretory progenitors into Paneth and goblet cells.^35^ In human cancers, SPDEF directly downregulated several oncogenes, including MMP9, Integrin-β3, and β-catenin, suppressing carcinogenesis.^14,16,36^ SPDEF promoted FOXM1’s promoter activity, inducing gastric tumor formation.^12^ However, in prostate cancer, SPDEF directly bound to the FOXM1 promoter and prevented its auto-regulatory activation, inhibiting carcinogenesis.^37^ Therefore, SPDEF can act as either an oncogene or a tumor suppressor in different cancers. Here, we found that SPDEF suppressed HNSCC cell proliferation, likely due to the effects of the tumor microenvironment or tumor cell stemness, and will be studied in our future work.

Dysregulation of the cell cycle is a hallmark of cancer, causing cells to divide indefinitely. Targeting the cell cycle has long been considered a promising anticancer measure.^38^ Lo et al.^15^ and Noah et al.^16^ reported that SPDEF could inhibit the occurrence of colorectal tumor by inducing cell cycle arrest. Schaefer et al.^39^ showed that SPDEF is a transcription factor that regulates p21/CIP1 expression, and proved for the first time that SPDEF is an inhibitor of breast tumor in vitro and in vivo systems. These studies showed that SPDEF could affect the occurrence and development of tumors by regulating cell cycle, which was consistent with the results of this study.

To explore the potential molecular mechanism of action of SPDEF in HNSCC, bioinformatics analysis and biochemistry experiments were conducted. We confirmed that SPDEF expression was positively correlated with NR4A1 expression and SPDEF could bind directly to the NR4A1 promoter to promote its transcription. NR4A1 inhibition reversed the tumor suppressive effects of SPDEF in HNSCC cells. NR4A1 has been reported to play critical roles in tumorigenesis by regulating several oncogenic signaling pathways, including acute myeloid leukemia, colorectal cancer, and oral squamous cell carcinoma.^18,21,25^ In acute myeloid leukemia, NR4A1/3-null hematopoietic stem cells showed elevated p-AKT and p-ERK1/2 levels and improved cell proliferation.^20^ Our results revealed that SPDEF suppressed p-AKT, p-GSK3β, and p-ERK1/2, demonstrating that SPDEF can inhibit several oncogenic signaling pathways by activating NR4A1 transcription. NF-κB signaling pathway suppression is one of the most common mechanisms of NR4A1 by directly interacting with the p65 subunit or by modulating the phosphorylation of p65.^40–43^ The NF-κB proteins are critical regulators of innate and adaptive immune responses which stimulate cell proliferation, inhibit apoptosis, accelerate cell migration and invasion, and promote angiogenesis and metastasis. The main tumorigenic effect of NF-κB is exerted through enhancement of cell proliferation and survival at the tumor promotion stage. By stimulating regeneration-enhancing inflammatory cytokines’ production, NF-κB enhances the proliferation of initiated tumor progenitor cells. NF-κB also increases the expression level of various cell cycle proteins, especially cyclin D1.^26^ Our results revealed that SPDEF suppressed p-p65 and its downstream genes, cyclin D1, IL1β, IL6, COX2, and GM-CSF by activating NR4A1 transcription.

Although we found that SPDEF might inhibit the malignant phenotype of HNSCC cells by activating NR4A1 transcription, there are still some limitations to the current study. Firstly, IHC analysis was conducted in a relatively small number of patients. A larger cohort should be used to further explore the relationship between SPDEF expression and HNSCC phenotypes. Secondly, the mechanism by which SPDEF is downregulated has not been explored. Further investigation is needed to elucidate this mechanism.

In summary, this study revealed a novel role for SPDEF in HNSCC. Downregulation of SPDEF in HNSCC was associated with poor clinical outcomes. SPDEF exerted its tumor suppressive effects by directly regulating the NR4A1/oncogenic signaling axis in HNSCC (Fig. 6f). Our findings provide novel insights into the mechanism of HNSCC tumorigenesis and a promising biomarker and therapeutic target for patients with HNSCC.

## Materials and methods

### Clinical specimen and cell lines

Clinical information and mRNA expression data for patients with HNSCC were downloaded from TCGA-HNSC dataset (http://gdc.cancer.gov) and GSE65858 dataset (http://www.ncbi.nlm.nih.gov/gds/). Data were retrieved on 20 October 2019. Human HNSCC tissues and adjacent non-cancerous epithelial tissues were collected from the Department of Craniofacial Surgery of the Hospital of Stomatology, Sun Yat-sen University. This experiment was approved by the Ethics Committee of the Hospital of Stomatology, Sun Yat-sen University, and informed consent was obtained from each patient.

The human HNSCC cell line CAL27 was purchased from ATCC (Manassas, VA, USA) and the cell line HSC6 was generously provided by J. Silvio Gutkind (National Institutes of Health, Bethesda, USA). Cells were cultured in Dulbecco’s Modified Eagle’s Medium (Gibco, USA) supplemented with 10% fetal bovine serum (Gibco, USA) at 37 °C in a humidified atmosphere containing 5% CO_2_. Cells were authenticated by PCR profiling using short tandem repeats.

### Real-time RT-PCR

Briefly, total RNA was isolated from tissues or cells using TRIzol (Invitrogen, Carlsbad, CA, USA) and then reverse-transcribed to synthesize cDNA. Real-time RT-PCR was conducted using ChamQ Universal SYBR qPCR Master Mix (Vazyme, China). Relative gene expression levels were normalized to GAPDH mRNA using the 2^−ΔΔCt^ method. The primers used in this study are listed in Table S4.

### Immunohistochemistry

IHC was performed on paraffin-embedded sections of human tissues and xenograft mouse tissues, as previously reported.^28^ The sections were deparaffinized and rehydrated using xylene and graded ethanol, respectively. Then, 3% hydrogen peroxide was applied to block endogenous horseradish peroxidase activity. Antigen retrieval was performed in a sodium citrate-hydrochloric acid buffer solution using microwaving samples. The sections were blocked with normal goat serum. Subsequently, the slides were incubated overnight at 4 °C with primary antibodies against SPDEF or NR4A1 and then with species-specific secondary antibodies. The antibodies used in this study are listed in Table S5.

Two experienced pathologists scored and validated all sections. Protein expression levels were calculated using the following equation: SI = staining intensity (0, no staining; 1, weak, light yellow; 2, moderate, yellow brown; 3, strong, brown) × percentage of positive cells (1, <10%; 2, 10%–35%; 3, 35%–70%; 4, >70%). Based on the SI, the staining pattern was defined as negative (SI = 0), weak (SI = 1–3), moderate (SI = 4–6), or strong (SI = 8-12).

### Western blotting assay

The cells were lysed using RIPA buffer supplemented with a protease inhibitor cocktail (CWBIO, China) and a phosphatase inhibitor cocktail (CWBIO, China). Protein extracts were separated using 10% sodium dodecyl sulfate polyacrylamide gel electrophoresis and then transferred onto polyvinylidene fluoride membranes (Millipore, USA). The membranes were blocked with 5% skimmed milk and incubated overnight at 4 °C with primary antibodies. The membranes were subsequently incubated with horseradish peroxidase-conjugated secondary antibodies. The immunoreactive bands were visualized using Immobilon Western Chemiluminescent HRP Substrate (Millipore, USA). In the silence assay of Figs. 2g and 4c, the immunoreactive bands were visualized using Immobilon ECL Ultra Western HRP Substrate (Millipore, USA). The antibodies used in this study are listed in Table S5.

### Small interfering RNAs and plasmids transfection

Negative control (NC) and siRNAs were synthesized by GenePharma (Shanghai, China). The sequences of the siRNAs targeting SPDEF and NR4A1 are listed in Table S4. Lipofectamine RNAiMAX transfection reagent (Invitrogen, USA) was used for transient transfection following the manufacturer’s instructions. The cells were collected 48 h after transfection with 50 nmol·L^-1^ siRNA oligonucleotides.

The pSin-EF2-puro-Vector and pSin-EF2-puro-SPDEF plasmids were obtained from Long Bioscience (China). Lentivirus packing plasmids were transfected into 293FT cells. Subsequently, the supernatants containing viruses were infected with HNSCC cells for 24–48 h. Stable clones were selected using 2 μg/mL puromycin (Solarbio, China). Infection efficiency was verified using real-time RT-PCR and western blotting assays.

### Cell viability assay, colony formation, and cell cycle analysis

For the cell viability assay, 1 000 cells per well were seeded into 96-well plates. At selected time points (1, 2, 3, 4, and 5 days), Cell Counting Kit-8 (CCK-8, Telenbiotech, China) was added into each well and incubated for 2 h following the manufacturer’s instructions. Absorbance was measured at 450 nm using a microplate reader.

For the colony formation assay, 500 cells per well were seeded into six-well plates and cultured for 1–2 weeks. The cells were then fixed with paraformaldehyde solution and stained with crystal violet.

For cell cycle analysis, cells were starved in serum-free medium for 24 h to allow them to synchronize. The cells were then incubated with medium supplemented with 10% FBS for a further 24 h. Then, 1 mL DNA staining solution and 10 µL permeabilization solution were used to resuspend and incubate the cells for 30 min at room temperature, following the instructions of the cell cycle kit manufacturer (Multi Sciences, China). Cell cycle distribution was determined using a flow cytometer (Beckman Coulter, USA).

### RNA sequencing and bioinformatic analysis

RNA-seq was used to identify genes that are transcriptionally regulated by SPDEF in HNSCC (Sinotech Genomics, China). Gene abundance was presented as fragments per kilobase of exon per million reads mapped. StringTie software was used to calculate the fragment within each gene, and the trimmed mean of M-value was used for normalization. Different expression analysis for mRNA was performed with R package edgeR. A *P* value < 0.05 and fold change value >1.5 were considered as significantly modulated and were obtained for further analysis. A heatmap was plotted in R software (https://www.r-project.org/). Gene Ontology (GO) analysis for biological processes and KEGG pathway analysis (http://www.genome.ad.jp/kegg) were performed using the enrich R package.

### Chromatin immunoprecipitation quantitative PCR

ChIP-qPCR was performed using a ChIP assay kit (ThermoFisher, USA) following the manufacturer’s protocol. Briefly, cells were cross-linked with 1% formaldehyde (Sigma, USA) at room temperature for 10 min and the reaction stopped with glycine. The nucleoprotein complexes were sheared to yield DNA fragments and immunoprecipitated with anti-HA or IgG (negative control) antibodies. Real-time RT-PCR was used to detect DNA fragment enrichment in the binding sites of the NR4A1 promoter. The primers used are listed in Table S4 while the antibodies used are listed in Table S5.

### Dual luciferase reporter assay

The pGL4-based luciferase reporter plasmids containing wild-type and mutant NR4A1 promoters were constructed by OBiO Technology (China). Cells were seeded in 24-well plates for 24 h and then co-transfected with pGL4-basic and Renilla luciferase using Lipofectamine 3000. A luciferase assay was performed using a dual luciferase reporter system (Promega, USA), following the manufacturer’s instructions. Luciferase activity was normalized to the Renilla activity value.

### Subcutaneous xenogeneic model

Five-week-old female BALB/c-nu mice were obtained from GemPharmatech (Nanjing, China) (*n* = 5 mice per group). All animal procedures were performed in accordance with the protocols approved by the Sun Yat-sen University Animal Research Committee. CAL27 cells stably overexpressing SPDEF or vector were suspended in serum-free medium. Next, 2 × 10^6^ cells were injected into the dorsal flank of the mice. The tumors were examined after 4 days, and then the tumor volumes measured every 3 days for 3 weeks. At the end of this period, the mice were killed and the tumors dissected and weighed.

### Statistical analysis

All statistical analyses were performed using SPSS software, and *P* < 0.05 was considered statistically significant. Data are presented as mean ± SD and are extracted from three independent experiments. Differences between groups were analyzed using Student’s *t* test or *χ*^2^ test. Mann–Whitney *U* test was used to analyze the protein level of SPDEF between the primary HNSCC tumors and adjacent non-cancerous epithelial tissues. Receiver operating characteristic curves were used to ascertain the optimal cutoff values for high and low SPDEF expression. Kaplan–Meier method was used to plot survival curves, and log-rank test was used to estimate survival rates. Bivariate correlations between variables were estimated using Spearman’s rank correlation coefficients.

## Supplementary information

supplementary materials

## Data Availability

The data used and/or analyzed during the current study are contained within the manuscript or available from the corresponding author on reasonable request.
