# Supplementary material for: SPDEF suppresses head and neck squamous cell carcinoma progression by transcriptionally activating NR4A1
Source: Int J Oral Sci. 2021 Oct 20;13:33. doi: 10.1038/s41368-021-00138-0 (PMC8526567; doi:10.1038/s41368-021-00138-0)
Supplement: Supplementary file 1 — supplementary materials [file 41368_2021_138_MOESM1_ESM.docx]

**Title Page**

**Research Article**

**Title:**

**SPDEF suppresses head and neck squamous cell carcinoma progression by transcriptionally activating NR4A1**

**Running Title:**

**Tumor suppression role of SPDEF in HNSCC**

**Authors and Affiliations:**

Yanting Wang^1,2,3^**^†^**, Xianyue Ren^1,2,3^**^†^**, Weiyu Li^1,2,3^, Ruoyan Cao^1,2,3^, Suyang Liu^1,2,3^, Laibo Jiang^1,2,3^, Bin Cheng^1,2,3^**^*^**, Juan Xia^1,2,3^**^*^**

^1^ Hospital of Stomatology, Sun Yat-sen University, Guangzhou, Guangdong, China

^2^Guangdong Provincial Key Laboratory of Stomatology, Guangzhou, Guangdong, China

^3^ Guanghua School of Stomatology, Sun Yat-sen University, Guangzhou, Guangdong, China

**Authors’ email addresses:**

wangyt69@mail2.sysu.edu.cn

[renxy7@mail.sysu.edu.cn](mailto:renxy7@mail.sysu.edu.cn)

[liwy95@mail2.sysu.edu.cn](mailto:liwy95@mail2.sysu.edu.cn)

[caory@mail2.sysu.edu.cn](mailto:caory@mail2.sysu.edu.cn)

liusy67@mail2.sysu.edu.cn

[jianglb3@mail2.sysu.edu.cn](mailto:jianglb3@mail2.sysu.edu.cn)

[chengbin@mail.sysu.edu.cn](mailto:chengbin@mail.sysu.edu.cn)

xiajuan@mail.sysu.edu.cn

**^†^Yanting Wang and Xianyue Ren contributed equally to this article.**

**^*^Corresponding author:**

**Juan Xia,** Guangdong Provincial Key Laboratory of Stomatology, Guanghua School of Stomatology, Sun Yat-sen University, Guangzhou 510055, Guangdong, People’s Republic of China; **Telephone:** +86 20 83880049; **Fax:** 020-83822807; **E-mail:** xiajuan@mail.sysu.edu.cn

**Bin Cheng**, Guangdong Provincial Key Laboratory of Stomatology, Guanghua School of Stomatology, Sun Yat-sen University, Guangzhou 510055, Guangdong, People’s Republic of China; **Telephone:** +86 20 83862558; **Fax:** 020-83822807; **E-mail:** chengbin@mail.sysu.edu.cn

**Figure S1**

**Figure S1 Quantitative analyses of western blots of SPDEF overexpression (a) and SPDEF knockdown (b).**


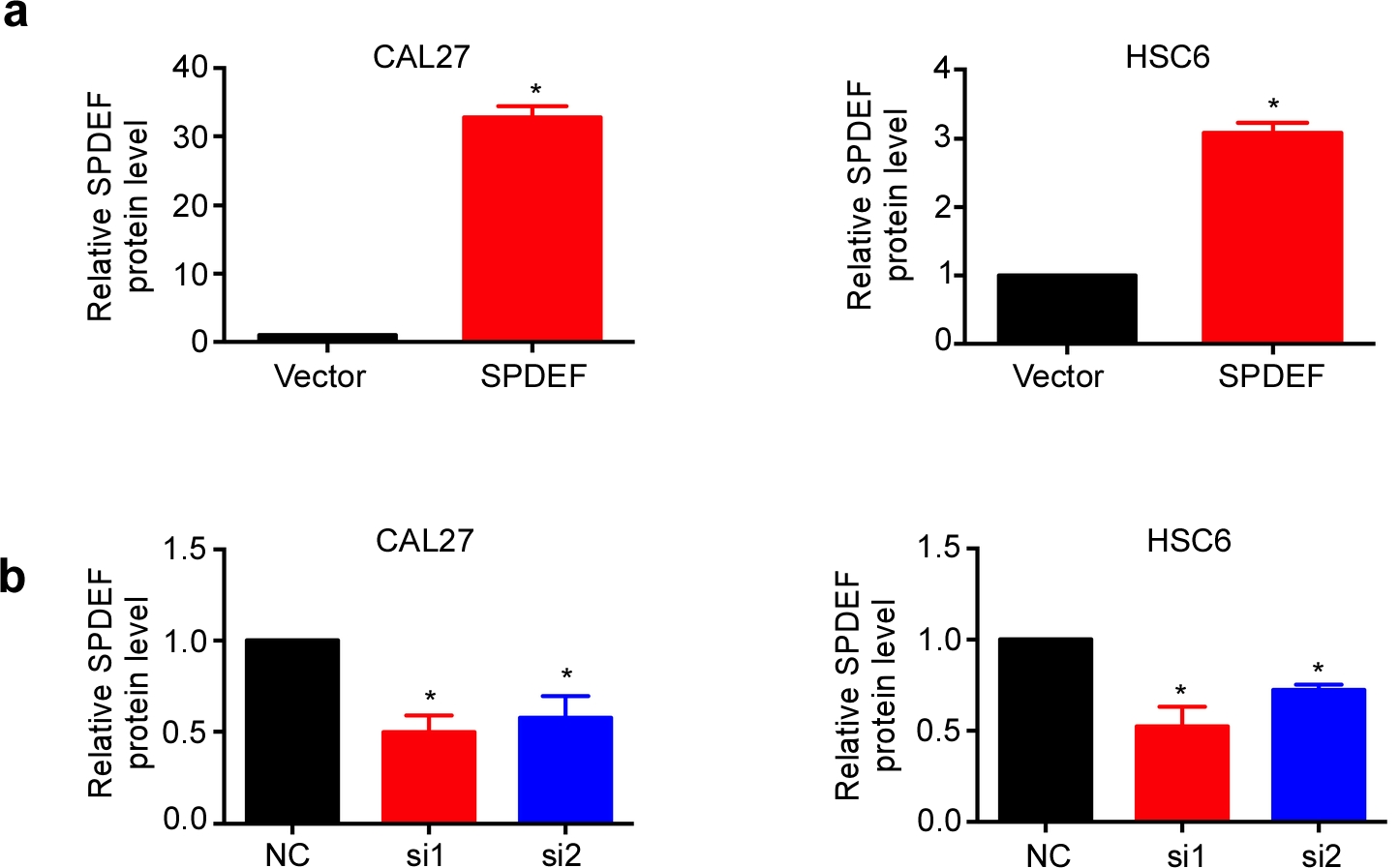


**Figure S2**

**Figure S2 The quantification of colonies was determined using the colony formation assay.**


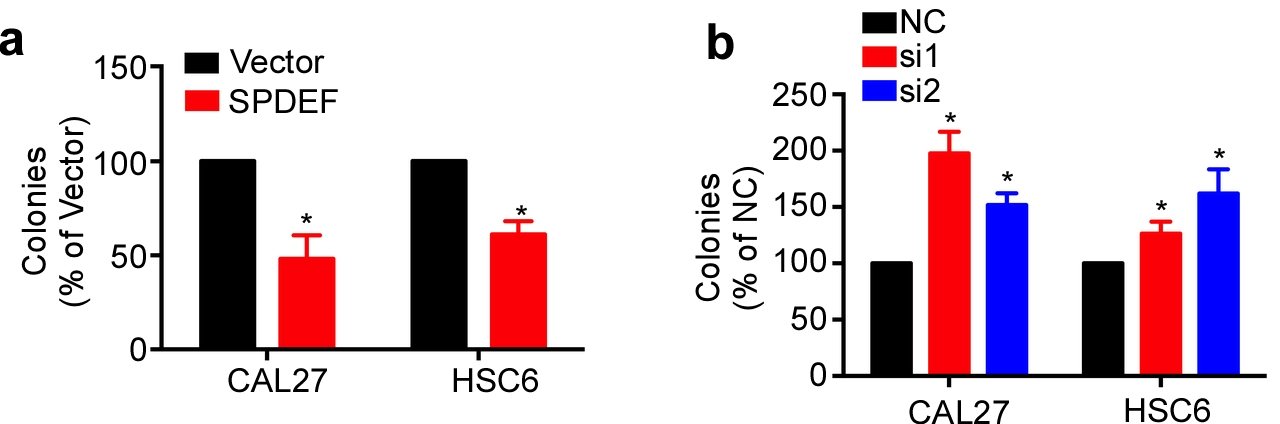


**Figure S3**

**Figure S3 Western blotting analysis of p-GSK3β and GSK3β protein levels. GAPDH was used as an endogenous control.**


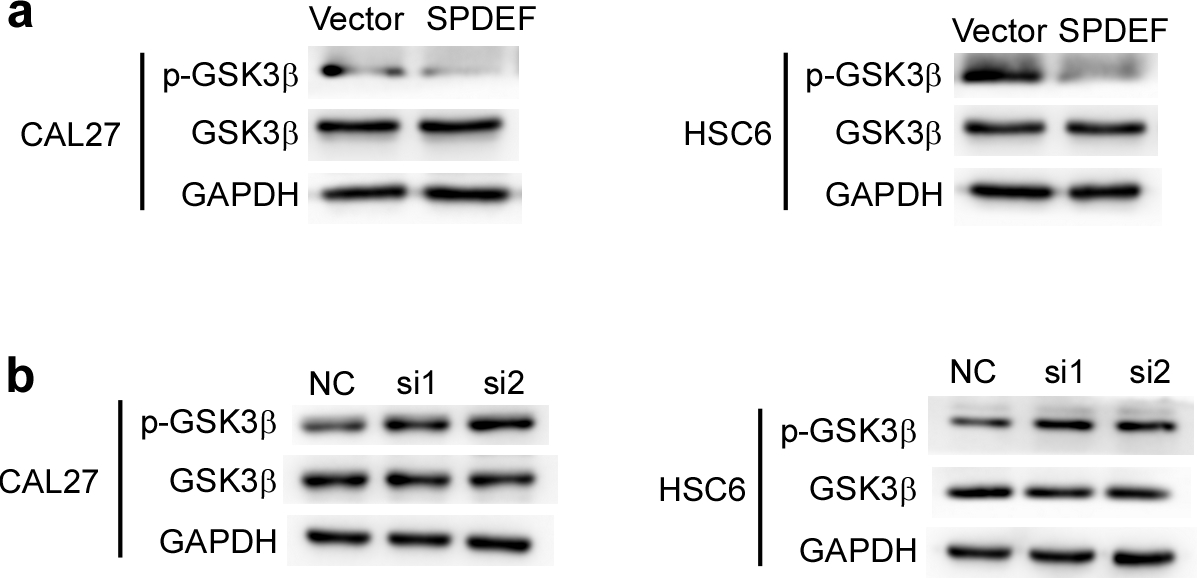


**Figure S4**

**Figure S4 Quantitative analyses of western blots of PI3K/AKT and MAPK signaling pathways.**


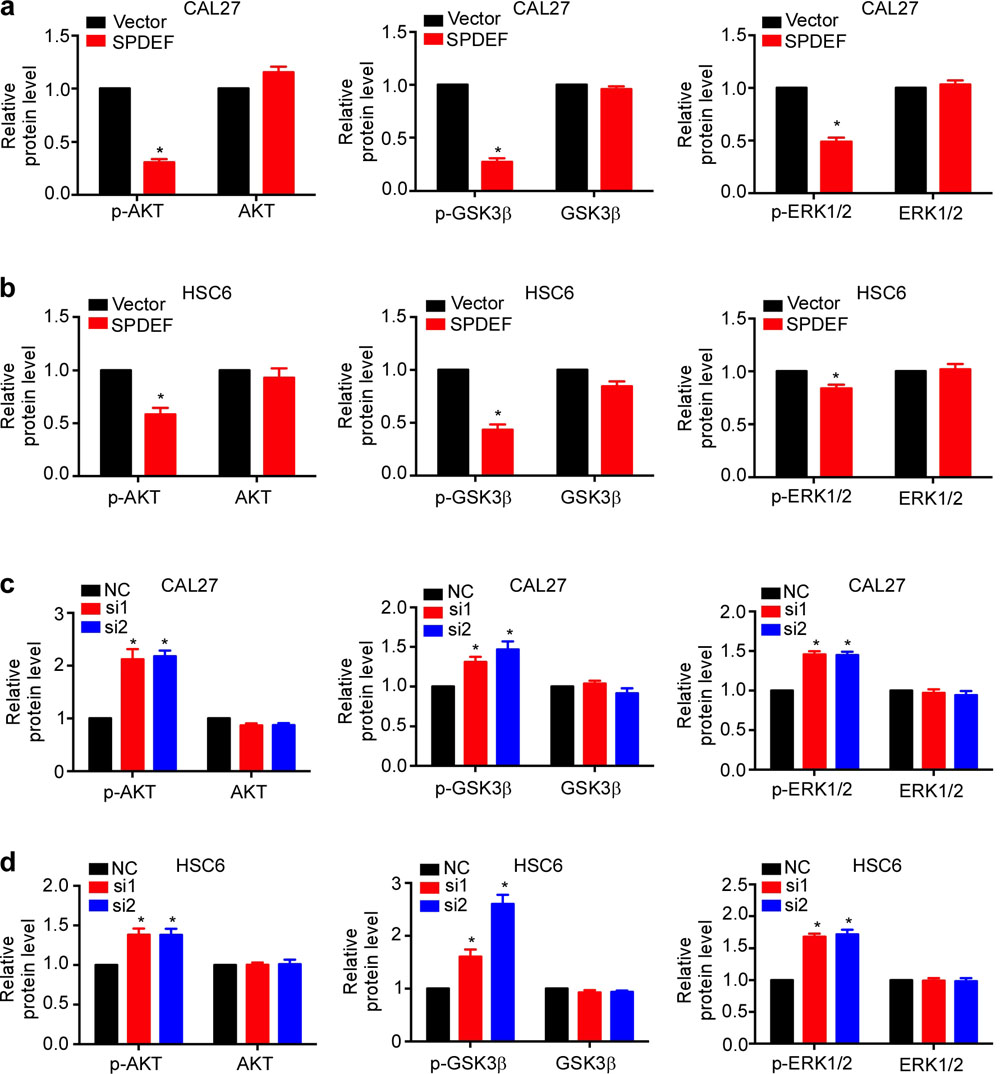


**Figure S5**

**Figure S5 Gene Ontology (GO) analysis and NF-κΒ targets’ expression of the RNA-seq data.** (a) GO analysis for biological processes of RNA-seq data. (b) NF-κB signaling target genes’ expression of the RNA-seq data.


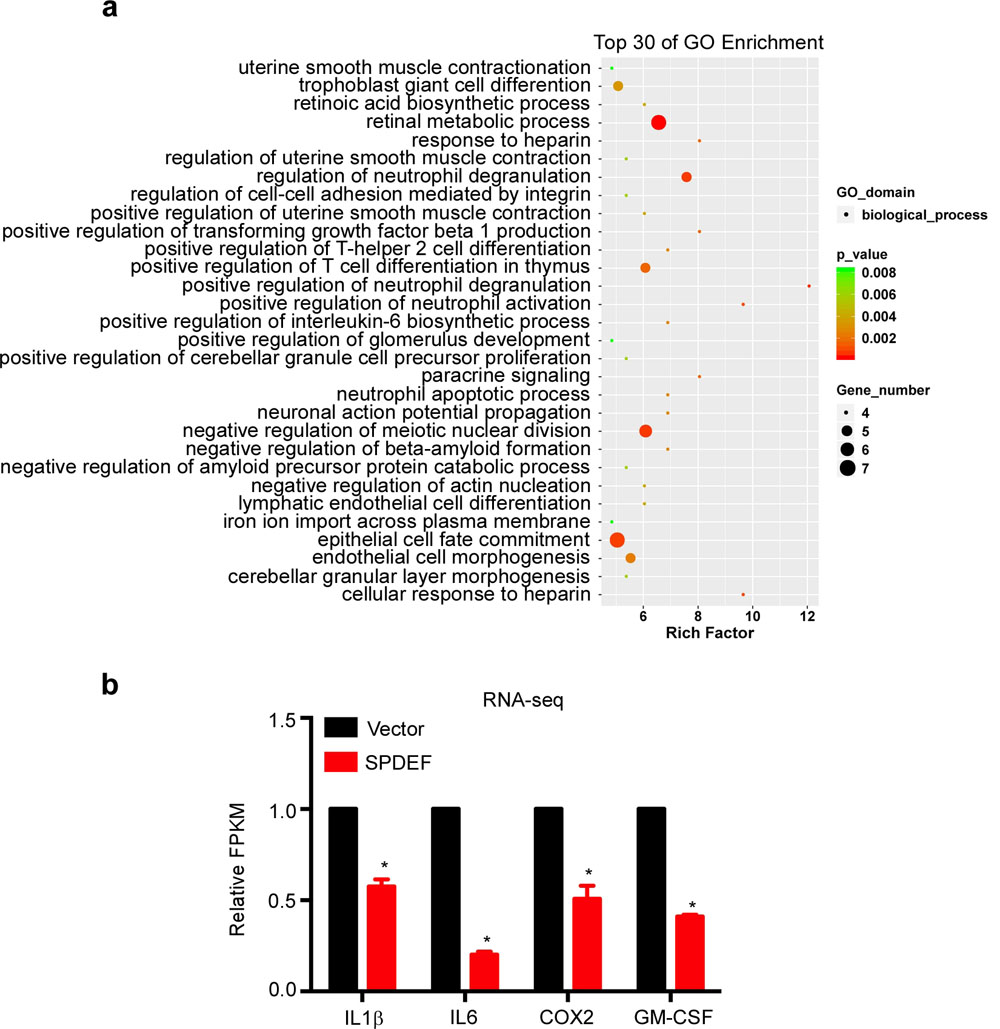


**Figure S6**

**Figure S6 Quantitative analysis of western blot of NF-κB signaling pathway.**


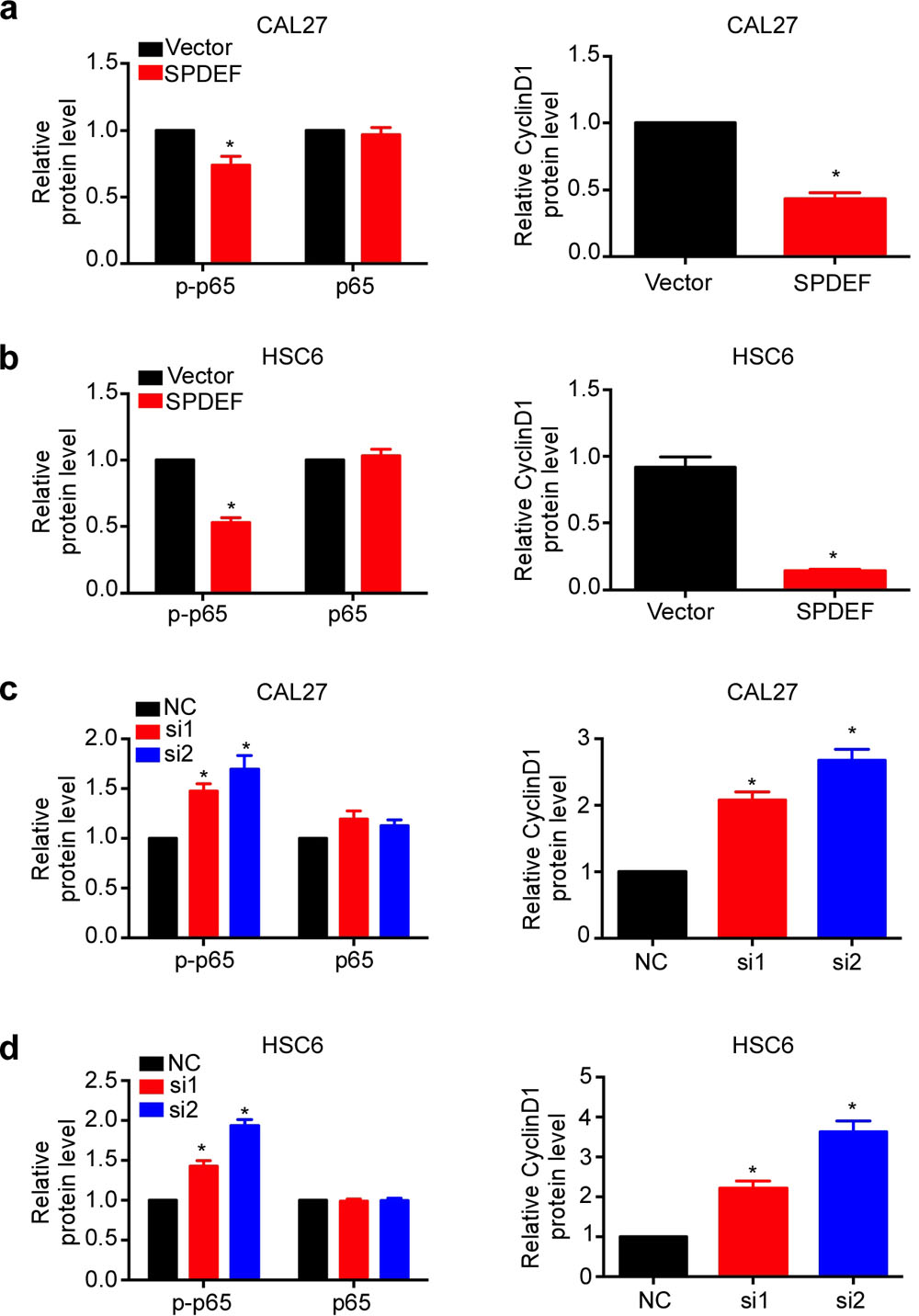


**Figure S7**

**Figure S7 Quantitative analyses of western blots of NR4A1 expression after SPDEF overexpression (a) or SPDEF knockdown (b).**


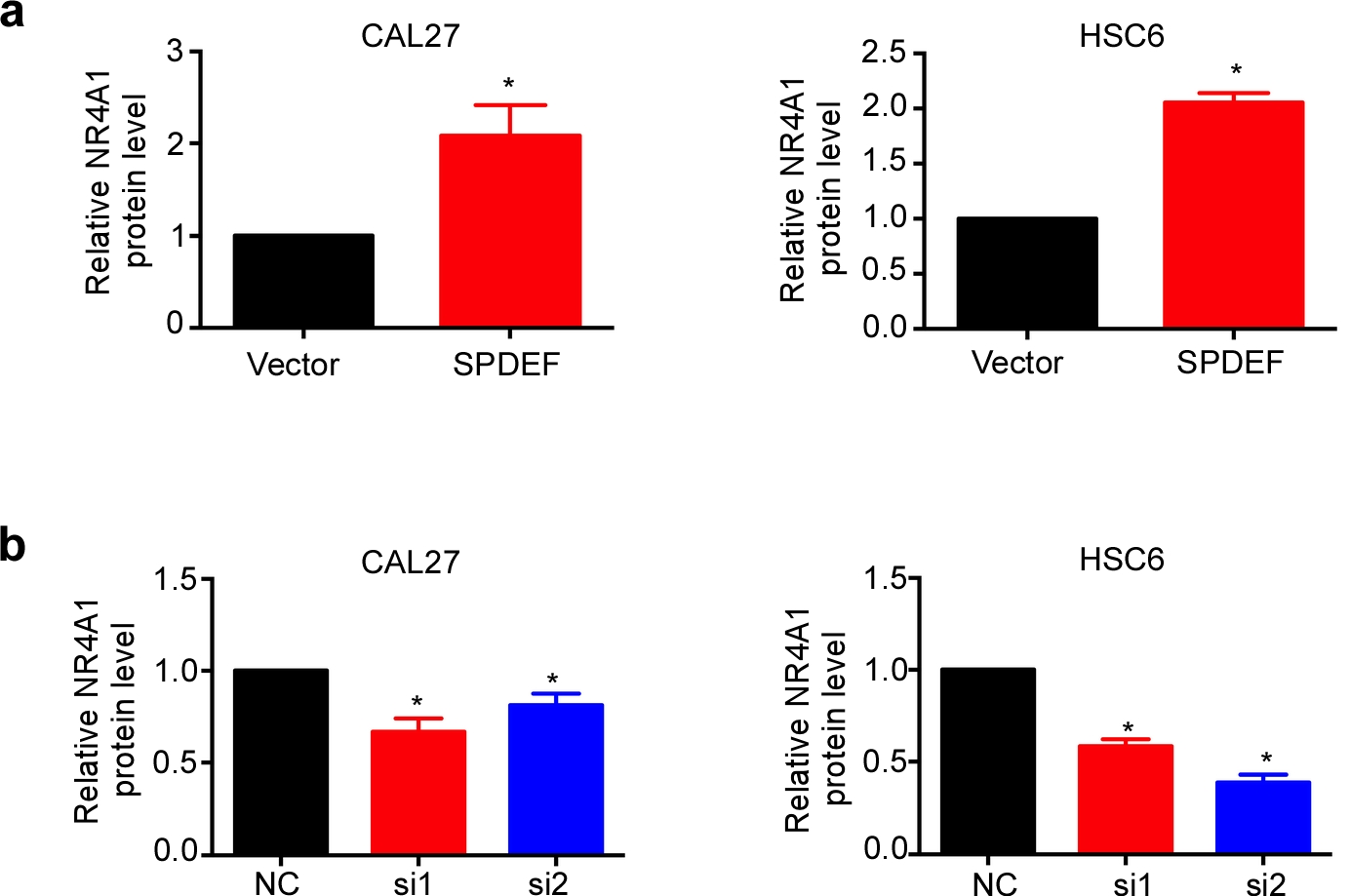


**Figure S8**

**Figure S8 SPDEF and NR4A1 levels were positively correlated in our hospital cohort.** (a) Correlation between SPDEF and NR4A1 mRNA levels in our cohort (n=17) was assessed using Spearman correlation analysis. (b–c) Correlation between SPDEF and NR4A1 protein levels in our cohort (n = 34) was assessed by IHC staining (×100 and ×200) and determined using Spearman correlation analysis. Scale bar: 40 µm.


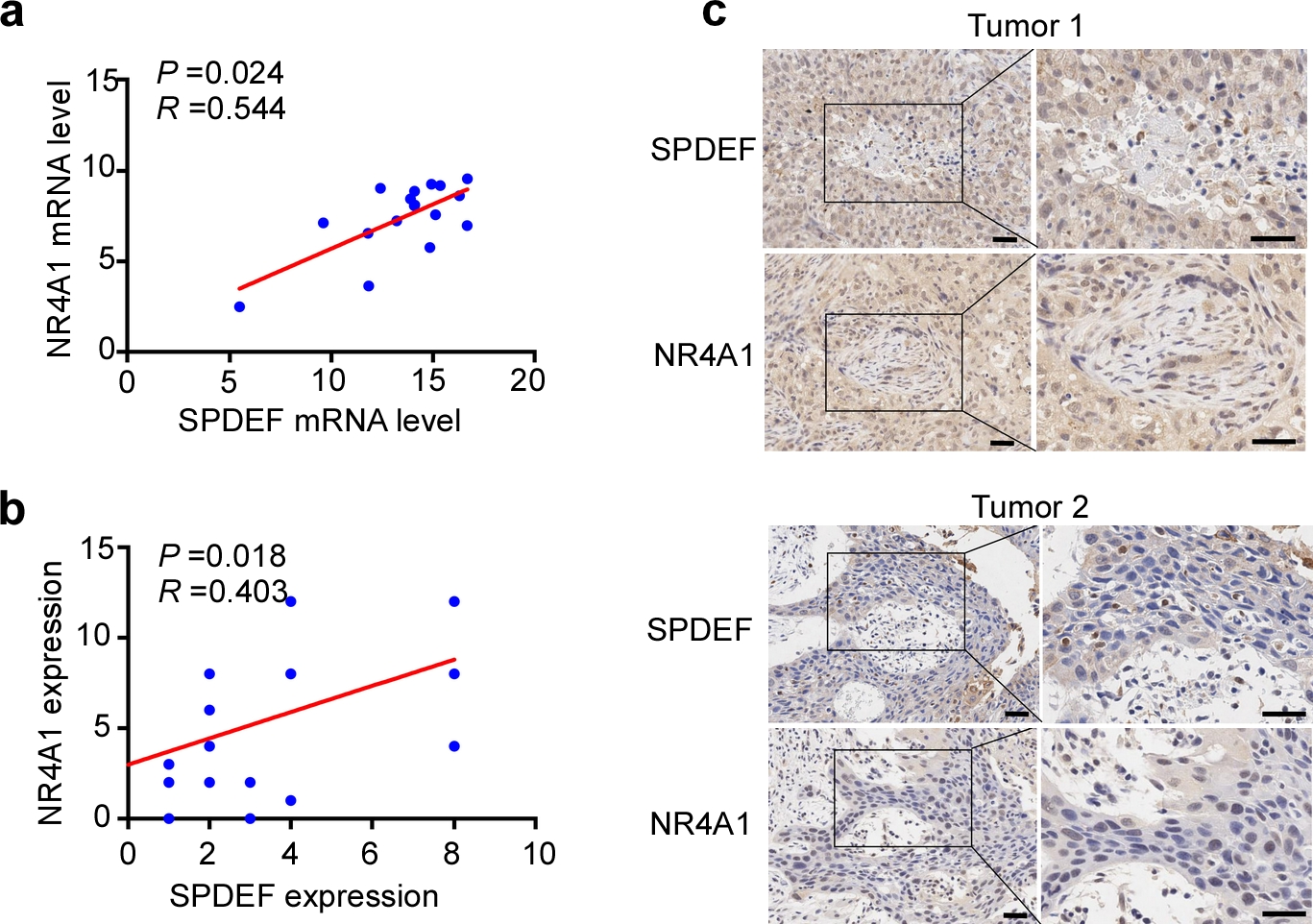


**Figure S9**

**Figure S9 Quantitative analysis of western blots of NR4A1 expression after silencing NR4A1.**


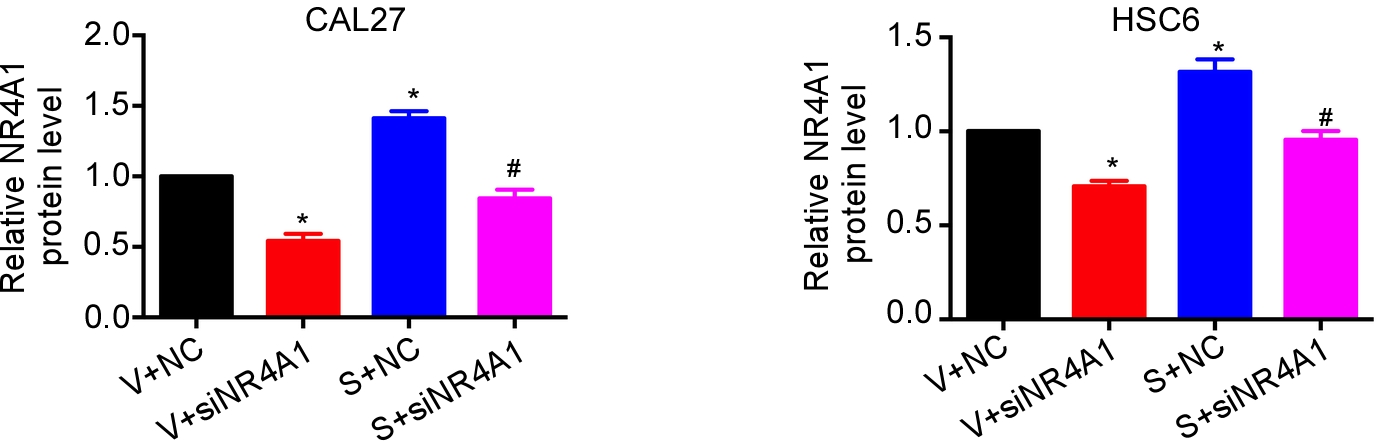


**Figure S10**

**Figure S10 The quantification of colonies was determined using the colony formation assay after silencing NR4A1.**


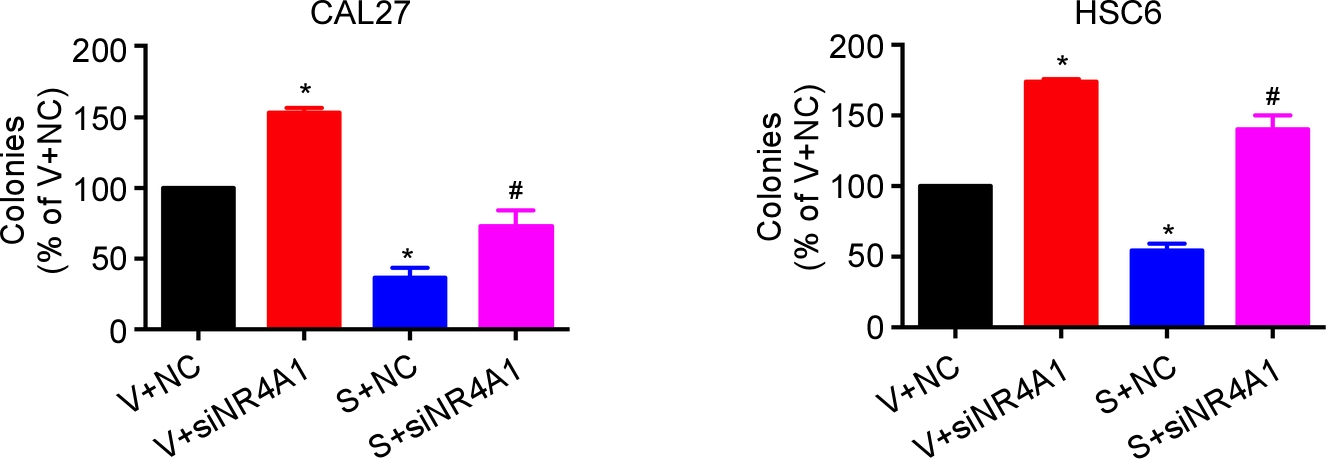


**Figure S11**


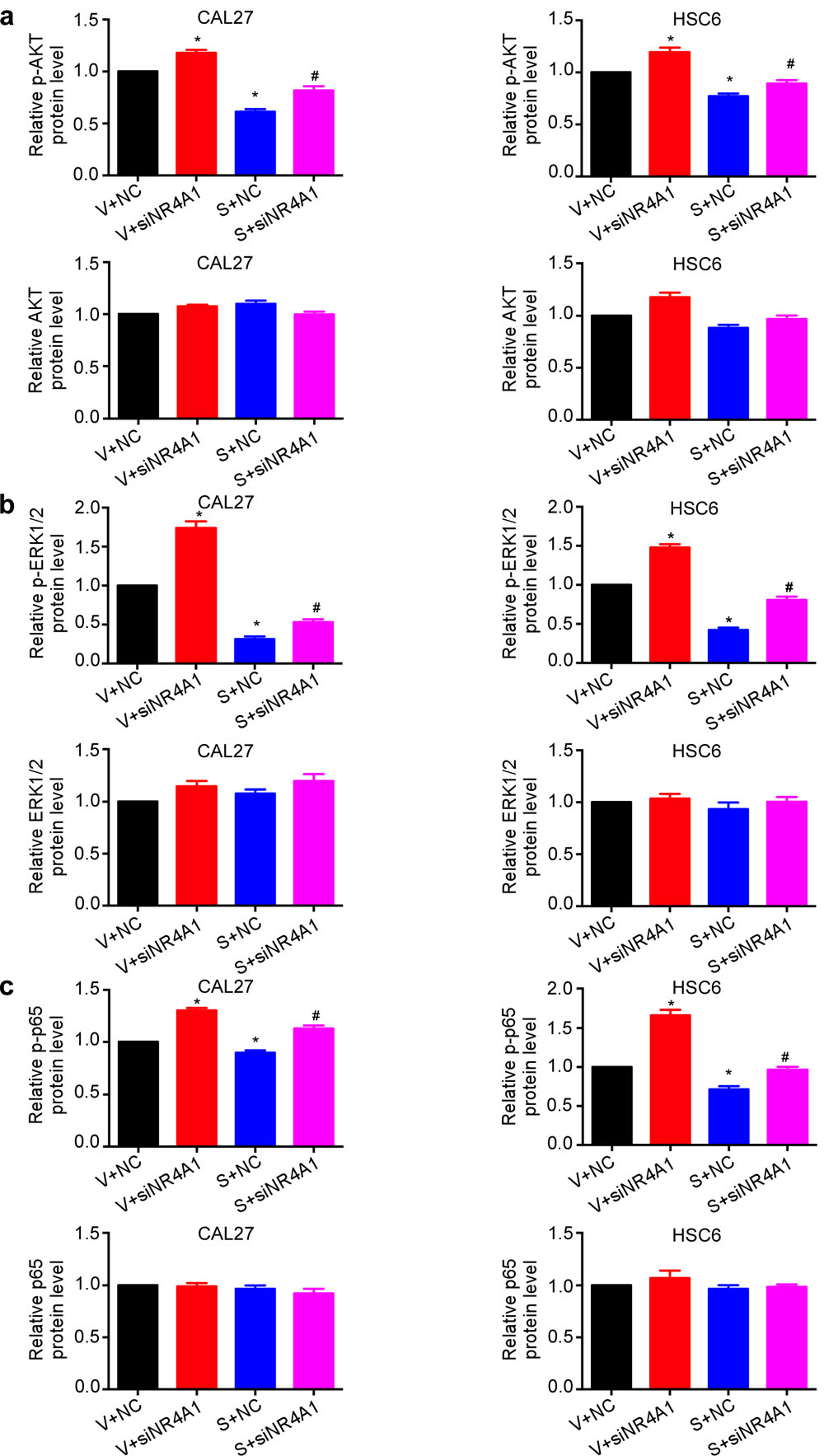


**Figure S11 Quantitative analysis of western blots of the activities of AKT (a), MAPK (b), and NF-κB (c) signaling pathways after silencing NR4A1.**

**Figure S12**

**Figure S12 Quantification analysis of SPDEF and NR4A1 protein levels and activities of AKT, MAPK, and NF-κB signaling pathways in mice xenografts were assessed by immunohistochemical staining.**


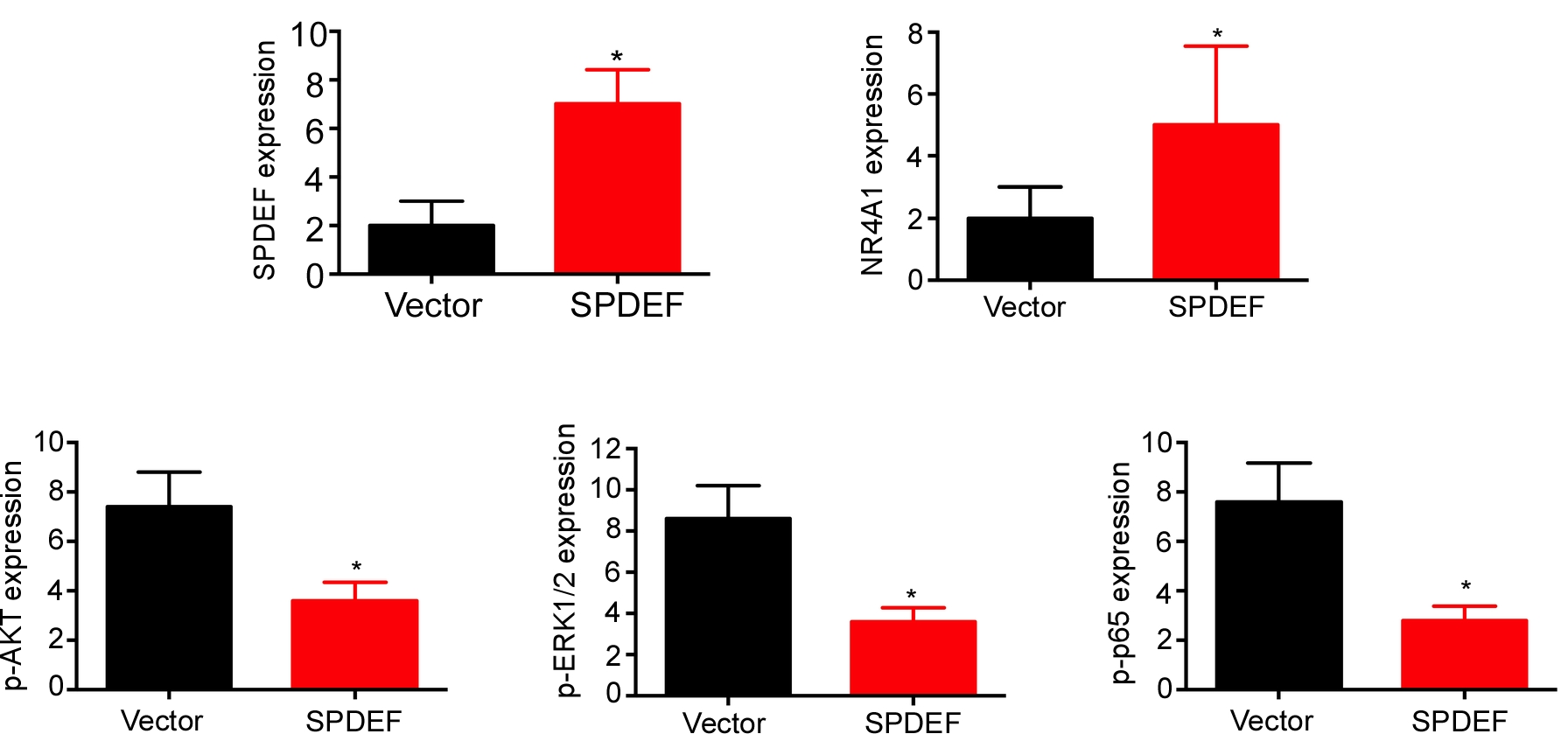


**Table S1 Relationship between SPDEF mRNA levels and clinicopathologic characteristics in TCGA-HNSC and GSE65858 cohorts.**

| Variables | TCGA-HNSC | | |  | GSE65858 | | |
| --- | --- | --- | --- | --- | --- | --- | --- |
|  | Low (%)  n =385 | High (%)  n =115 | *P* value^a^ |  | Low (%)  n =146 | High (%)  n =124 | *P* value^a^ |
| Age |  |  |  |  |  |  |  |
| <60 years | 171 (78.4) | 47 (21.6) | 0.716 |  | 80 (52.3) | 73 (47.7) | 0.501 |
| ≥60 years | 213 (76.3) | 66 (23.7) |  |  | 66 (56.4) | 51 (43.6) |  |
| Gender |  |  |  |  |  |  |  |
| Male | 280 (76.3) | 87 (23.7) | 0.533 |  | 115 (51.6) | 108 (48.4) | 0.072 |
| Female | 105 (78.9) | 28 (21.1) |  |  | 31 (66.0) | 16 (34.0) |  |
| T stage |  |  |  |  |  |  |  |
| T1-T2 | 143 (80.8) | 34 (19.2) | 0.105 |  | 50 (43.5) | 65 (56.5) | 0.003* |
| T3-T4 | 198 (74.2) | 69 (25.4) |  |  | 96 (61.9) | 59 (38.1) |  |
| N stage |  |  |  |  |  |  |  |
| N_0_ | 127 (74.3) | 44 (25.7) | 0.332 |  | 48 (51.1) | 46 (48.9) | 0.468 |
| N_1_ - N_3_ | 185 (78.4) | 51 (21.6) |  |  | 98 (55.7) | 78 (44.3) |  |
| TNM stage |  |  |  |  |  |  |  |
| I-II | 78 (82.1) | 17 (17.9) | 0.123 |  | 24(45.3) | 29 (54.7) | 0.152 |
| III-IV | 251 (76.3) | 86 (83.5) |  |  | 122 (56.2) | 95 (43.8) |  |

^a^χ^2^ test. **P* < 0.05

**Table S2 Top 20 genes identified from ChIP-seq, RNA-seq, and Spearman correlation analyses.**

| **Gene name** | ***R*** | ***P*** |
| --- | --- | --- |
| C9orf152 | 0.546982 | 0 |
| ARHGEF38 | 0.43616 | 0 |
| DMBT1 | 0.436022 | 0 |
| KRT7 | 0.403438 | 0 |
| KIAA1324 | 0.397895 | 0 |
| GCNT3 | 0.371248 | 0 |
| MUC1 | 0.339585 | 1.78E-15 |
| ELF3 | 0.273044 | 2.42E-10 |
| PTPRU | 0.221235 | 3.47E-07 |
| SCNN1A | 0.220078 | 4E-07 |
| NPTXR | 0.210798 | 1.23E-06 |
| FAM107B | 0.198849 | 4.89E-06 |
| ALCAM | 0.192931 | 9.4E-06 |
| RTBDN | 0.191621 | 1.08E-05 |
| KAZALD1 | 0.181545 | 3.12E-05 |
| LRRC6 | 0.177026 | 4.92E-05 |
| GIPR | 0.17073 | 9.13E-05 |
| SNCG | 0.162235 | 0.000203 |
| NR4A1 | 0.155598 | 0.000369 |
| SRGAP3 | 0.153771 | 0.000433 |

**Table S3 Four potential candidate binding sites identified in the NR4A1 promoter region.**

| **Name** | **Start** | **End** | **Gene ID** | **Symbol** |
| --- | --- | --- | --- | --- |
| SPDEF | 923 | 933 | 3164 | NR4A1 |
| SPDEF | 1123 | 1133 | 3164 | NR4A1 |
| SPDEF | 1604 | 1614 | 3164 | NR4A1 |
| SPDEF | 753 | 763 | 3164 | NR4A1 |

**Table S4 Primers used in this study.**

| **Gene** | **Sequence (5' to 3')** |
| --- | --- |
| **Real-time RT-PCR primers** | |
| SPDEF-F | CAGTGCCCGGTCATTGACA |
| SPDEF-R | CAGCCGGTATTGGTGCTCT |
| NR4A1-F | CCCTGAAGTTGTTCCCCTCAC |
| NR4A1-R | GCCCTCAAGGTGTGGAGAAG |
| IL1β-F | ATGATGGCTTATTACAGTGGCAA |
| IL1β-R | GTCGGAGATTCGTAGCTGGA |
| IL6-F | ACTCACCTCTTCAGAACGAATTG |
| IL6-R | CCATCTTTGGAAGGTTCAGGTTG |
| COX2-F | CTGGCGCTCAGCCATACAG |
| COX2-R | CGCACTTATACTGGTCAAATCCC |
| GM-CSF-F | TCCTGAACCTGAGTAGAGACAC |
| GM-CSF-R | TGCTGCTTGTAGTGGCTGG |
| GAPDH-F | GAGTCAACGGATTTGGTCGT |
| GAPDH-R | GACAAGCTTCCCGTTCTCAG |
| **siRNA sequences** | |
| siRNA-NC-F | UUCUCCGAACGUGUCACGUTT |
| siRNA-NC-R | ACGUGACACGUUCGGAGAATT |
| siSPDEF-1-F | GCGAAGUGCUCAAGGACAUTT |
| siSPDEF-1-R | AUGUCCUUGAGCACUUCGCTT |
| siSPDEF-2-F | CCCGGUCAUUGACAGCCAATT |
| siSPDEF-2-R | UUGGCUGUCAAUGACCGGGTT |
| siNR4A1-F | CAGUCCAGCCAUGCUCCUCTT |
| siNR4A1-R | GAGGAGCAUGGCUGGACUGTT |
| **ChIP-qPCR primers** | |
| NR4A1-1-F | AGGCTCAGGAGAGATCAGGGTGGAA |
| NR4A1-1-R | GCTCTGACCAGTTATCACCTGCCCG |
| NR4A1-2-F | GGGCAGGTGATAACTGGTCAGAGCT |
| NR4A1-2-R | AGGTGGTGGCACACTGGGTTGGAAC |
| NR4A1-3-F | CAGGCTCCACCCGGTTCTGAAATTC |
| NR4A1-3-R | AACAGCTCTGGCTCCGCTCCACAAG |
| NR4A1-4-F | CTCCAGGAAGGGCTTGGGAAGGTGT |
| NR4A1-4-R | AGAATAACCAGCGGGAGGGCCAGAG |

**Table S5 Antibodies information.**

| **Reagent** | **Company** | **Catalog No.** | **Application** |
| --- | --- | --- | --- |
| SPDEF | Abcam | Ab53881 | WB, 1:1000 |
| SPDEF | Abcam | Ab197375 | IHC, 1:50 (human) |
| SPDEF | Abcam | Ab197375 | IHC, 1:100 (mice) |
| Phospho-Akt (Ser 473) | Cell Signaling Technology | 4060 | WB, 1:1000 |
| Phospho-Akt (Ser 473) | Cell Signaling Technology | 4060 | IHC, 1:50 (mice) |
| Akt | Cell Signaling Technology | 4685 | WB, 1:1000 |
| Phospho-GSK-3β(Ser9) | Cell Signaling Technology | 9336 | WB, 1:1000 |
| GSK-3β | Cell Signaling Technology | 12456 | WB, 1:1000 |
| Phospho-p44/42 MAPK (Erk 1/2) | Cell Signaling Technology | 4370 | WB, 1:1000 |
| Phospho-p44/42 MAPK (Erk 1/2) | Cell Signaling Technology | 4370 | IHC, 1:100 (mice) |
| p44/42 MAPK (Erk 1/2) | Cell Signaling Technology | 4695 | WB, 1:1000 |
| NF-kB p65 | Cell Signaling Technology | 8242 | WB, 1:2000 |
| Phospho-NF-kB p65  (Ser536) | Cell Signaling Technology | 3033 | WB, 1:2000 |
| Phospho-NF-kB p65  (Ser536) | Cell Signaling Technology | 3033 | IHC, 1:100 (mice) |
| NR4A1 | Cell Signaling Technology | 3960 | WB, 1:1000 |
| NR4A1 | Santa Cruz | sc-365113 | IHC, 1:200 (human) |
| NR4A1 | Santa Cruz | sc-365113 | IHC, 1:100 (mice) |
| Cyclin D1 | Cell Signaling Technology | 55506 | WB, 1:1000 |
| GAPDH | Proteintech | 60004-1-Ig | WB, 1:4000 |
| HA-Tag | Cell Signaling Technology | 3724 | ChIP, 1:50 |
